# Morphofunctional Profile Focusing on Strength and Ultrasound of the Upper Limbs in Female Breast Cancer Survivors: A Comparative Cross-Sectional Study Between Groups with and Without Lymphoedema and Between Ipsilateral and Contralateral Limbs

**DOI:** 10.3390/biomedicines13081884

**Published:** 2025-08-02

**Authors:** Ana Rafaela Cardozo Da Silva, Juliana Netto Maia, Vanessa Maria Da Silva Alves Gomes, Naiany Tenório, Juliana Fernandes de Souza Barbosa, Ana Claudia Souza da Silva, Vanessa Patrícia Soares de Sousa, Leila Maria Alvares Barbosa, Armèle de Fátima Dornelas de Andrade, Diego Dantas

**Affiliations:** 1 Department of Physical Therapy, Health Sciences Center, Federal University of Pernambuco, Recife 50670-901, Brazil; rafaela.cardozo@ufpe.br (A.R.C.D.S.); juliana.netto@ufpe.br (J.N.M.); vanessaalvesfta@gmail.com (V.M.D.S.A.G.); n.tenorio.j@gmail.com (N.T.); juliana.fsbarbosa@ufpe.br (J.F.d.S.B.); claudia.souzasilva@ufpe.br (A.C.S.d.S.); leila.barbosa@ufpe.br (L.M.A.B.); armele.andrade@ufpe.br (A.d.F.D.d.A.); 2 Faculty of Health Sciences of Trairi, Federal University of Rio Grande do Norte, Santa Cruz 59200-000, Brazil; vanessa.sousa@ufrn.br

**Keywords:** muscle strength, breast cancer-related lymphoedema, breast neoplasms, ultrasound

## Abstract

**Background**: Breast cancer is the most common neoplasm in women. Despite effective treatments, sequelae such as decreased muscle strength, upper limb dysfunction, and tissue changes are common, highlighting the need for functional assessments during rehabilitation. This study analysed the morphofunctional profile of the upper limbs in breast cancer survivors, comparing muscle strength and ultrasound findings between groups with and without lymphoedema, as well as between ipsilateral and contralateral limbs. **Methods**: This cross-sectional study included female breast cancer survivors treated at an oncology physical therapy clinic. Muscle strength was measured using dynamometry (handgrip and arm flexor strength), and ultrasound assessed the thickness of the dermal–epidermal complex (DEC), subcutaneous tissue (SUB), and muscle (MT). **Results**: The upper limbs of 41 women were evaluated. No significant differences were observed between those with and without breast cancer-related lymphoedema (BCRL). When comparing the ipsilateral and contralateral limbs, significant reductions were observed in arm flexor strength (*p* < 0.001; 95% CI: −9.77 to −2.50), handgrip strength (*p* < 0.001; 95% CI: −4.10 to −1.22), and tissue thickness, with increased DEC thickness on the forearm (0.20 mm; *p* = 0.022) and arm flexors (0.25 mm; *p* < 0.001) of the ipsilateral limb. **Conclusion**: Significant differences in muscle strength and tissue structure between ipsilateral and contralateral limbs may reflect surgical and local pathophysiological effects. A trend toward reduced values for these parameters was also noted in limbs with BCRL, reinforcing the importance of future research to elucidate underlying mechanisms and guide more effective therapeutic strategies.

## 1. Introduction

Breast cancer is the most prevalent malignancy among women, accounting for approximately one in every four cancer diagnoses and one in every six cancer-related deaths [1]. In Brazil, an estimated 73,610 new cases are projected for the 2023–2025 period, according to the National Cancer Institute [2], underscoring the disease’s magnitude and impact.

Surgery remains a cornerstone of breast cancer treatment, significantly improving survival rates [3]. However, advances in therapeutic strategies and the resulting increase in survival rates have drawn greater attention to the long-term adverse effects of interventions such as surgery, chemotherapy, and radiotherapy [4]. The most common complications include lymphoedema, fatigue, upper limb dysfunction, and reduced muscle strength and cardiorespiratory fitness [4,5].

While essential for disease control, chemotherapy can impair muscle metabolism, leading to a loss of muscle mass and strength, particularly in the upper limbs, and a decline in overall physical fitness [6]. Radiotherapy, in turn, can indirectly alter muscle morphology, causing reductions in muscle volume and length and resulting in significant functional impact [7].

These musculoskeletal impairments can be acute or chronic, leading to dysfunctions such as reduced range of motion, decreased muscle strength, postural changes, and limitations in the activities of daily living (ADLs) [8].

These changes often lead to chronic pain and discomfort in the upper limbs, regardless of the treatment modality [9]. Beyond the physical repercussions, these alterations adversely affect quality of life, leading to significant psychosocial consequences such as symptoms of anxiety and depression and reduced social participation [10].

Functional impairment affects multiple dimensions of quality of life, including general health perception, physical performance, social roles, emotional state, body image, sexuality, and sexual satisfaction [11]. Despite the clinical relevance of complications related to muscle dysfunction, there is limited literature on the detailed characterisation of tissue and functional muscle changes in breast cancer survivors.

The early detection of morphofunctional changes is crucial. Combining objective methods, such as muscle strength assessment and tissue ultrasound, enables a more comprehensive analysis of patients’ morphofunctional profiles. This contributes to preventing and managing complications and guiding individualised rehabilitation strategies [12]. In this context, precise assessments are essential for preserving functionality and quality of life in female breast cancer survivors, allowing for early identification of patients at higher risk of morbidities and for the implementation of more effective therapeutic interventions [7,13].

This study aims to analyse the morphofunctional profile of the upper limbs in breast cancer survivors by comparing muscle strength and ultrasound findings between groups with and without lymphoedema, as well as between the ipsilateral and contralateral limbs. Integrating assessments of muscle function and tissue structure improves the clinical monitoring of these patients by providing a deeper understanding and enabling the early detection of functional changes. It can also guide interventions aimed at preserving functionality and improving quality of life.

## 2. Patients and Methods

### 2.1. Study Population

This cross-sectional study followed the *Strengthening the Reporting of Observational Studies in Epidemiology* (STROBE) guidelines [14]. The research protocol was approved by the Institutional Ethics Committee (CAAE: 57624121.0.0000.5208), and all participants provided written informed consent.

This study was conducted from August 2022 to September 2023 at the Laboratory of Physical Therapy in Women’s Health and Pelvic Floor (LAFISMA), Department of Physical Therapy, Federal University of Pernambuco (UFPE).

The study population comprised female breast cancer survivors residing in Recife who were treated at LAFISMA. Inclusion criteria were women aged 40 to 70 with a history of mastectomy. Exclusion criteria included primary lymphoedema, oedema from other causes (rheumatologic, renal, or neurologic disease; orthopaedic issues; or prior vascular disease), skin conditions (erysipelas, intertrigo, or ulcers), undergoing chemotherapy or radiotherapy, and left-handedness (to ensure sample homogeneity).

Participants were recruited consecutively from the waiting list for oncology physical therapy care.

The sample size was calculated using G*Power 3.1 software (version 3.1, Düsseldorf, Germany) for a comparison between two independent groups (Student’s *t*-test). The effect size (d = 0.92) was estimated based on a previous study by Perez et al. that found a significant reduction in handgrip strength on the ipsilateral limb in women with breast cancer [15]. With a statistical power of 80% and an alpha of 0.05, accounting for a potential 20% sample loss or incomplete data, a minimum sample size of 39 women was required.

### 2.2. Data Collection

Data were collected at the LAFISMA facility by trained and calibrated assessors. The assessors underwent three four-hour theoretical and practical training sessions conducted by an experienced physical therapist. The training covered all study assessment procedures, including taking a medical history, performing a lymphoedema assessment, conducting an ultrasound examination, and measuring muscle strength.

Initially, participants completed a clinical form to collect personal and clinical information, including age (years), marital status, education level, time since surgery (months), type of mastectomy, and treatments received. Breast cancer-related lymphoedema (BCRL) was assessed according to the International Society of Lymphology guidelines [16]. A BCRL diagnosis was confirmed if the difference in upper limb volume was ≥200 mL or if the volume ratio was >1.04, as determined by indirect volumetry [17].

Muscle strength assessment included measurements of arm flexor muscle strength (AFMS) and absolute and relative handgrip strength (HGS). HGS was measured using a calibrated hydraulic hand dynamometer (Carci, São Paulo, Brazil). For the HGS measurement, participants were seated with their shoulder adducted and neutrally rotated, elbow flexed at 90°, forearm in a neutral position, and wrist at 0° and 30° of extension and 0° to 15° of ulnar deviation. The contralateral limb was relaxed on the thigh [18].

Assessors verbally instructed participants to exert maximal voluntary isometric force. Three sustained contractions were performed, with a one-minute rest interval between measurements [19]. Absolute HGS (AHGS) was defined as the average of the three measurements, expressed as a continuous variable in kilograms (kg). Relative HGS (RHGS) was calculated by normalising the AHGS value by the body mass index (BMI). RHGS was presented in kg/m^2^ [20,21].

AFMS was assessed using a portable digital dynamometer (Lafayette Hand-Held Dynamometer, Lafayette, LA, USA), which has demonstrated excellent test–retest and intra-rater reliability [22]. During the assessment, the participant was instructed to exert maximal elbow flexion force against resistance applied by the assessor at a 90° angle (±5°) to the participant’s arm. The average of three trials was recorded for mean force (N), peak force (N), and time to failure (seconds). The test was stopped when the participant could no longer maintain resistance [22].

**Figure 1:**
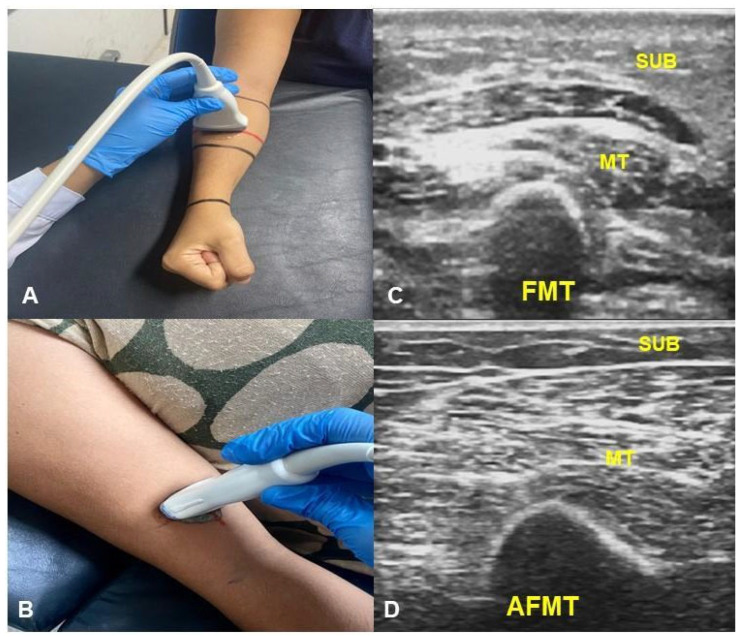
(**A**) Patient and transducer positioning for ultrasound imaging of forearm flexors (FMT); (**B**) Ultrasound image of FMT; (**C**) Patient and transducer positioning for ultrasound imaging of arm flexors (AFMT); (**D**) ultrasound image of AFMT. SUB: subcutaneous tissue; MT: muscle thickness. Source: The authors (2005).

**Figure 2:**
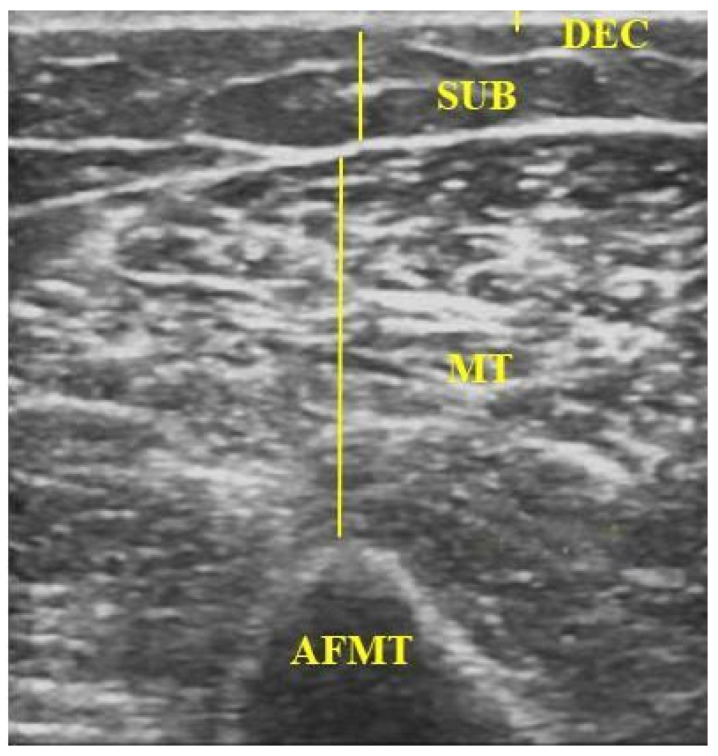
Ultrasound image showing measurements of DEC, SUB, and MT of the arm flexor muscles. DEC: dermal–epidermal complex; SUB: subcutaneous tissue; MT: muscle thickness; AFMT: arm flexor muscle thickness. Source: the authors (2025).

B-mode ultrasound images were acquired using a LOGIC V5 system (GE) with a linear L6-12 transducer. Standardised parameters were used for all measurements: 8 MHz frequency, 79% gain, and 8 cm depth. In each limb, two regions of interest (ROIs) were defined, one on the forearm and one on the arm, both 10 cm from the cubital fossa [23]. Images were captured with the transducer positioned perpendicular to the skin over the muscle belly of interest (Figure 1), applying minimal pressure and using a water-based conductive gel [24]. After freezing the image, the following were measured: (i) the dermal–epidermal complex (DEC)—the linear distance from the posterior echogenic border of the epidermal entry echo to the posterior echogenic border of the dermis [25]; (ii) subcutaneous tissue (SUB)—the distance between the DEC and the muscle fascia [25,26]; (iii) muscle thickness (MT) of the forearm flexors (FMT)—the distance between the radius and the muscle interface [27]; and muscle thickness of the arm flexors (AFMT)—the distance between the muscle interface and the humeral periosteum, including the biceps brachii and brachialis muscles (Figure 2) [28,29].

For the forearm assessment, the upper limb was supported on a table and positioned with the shoulder flexed (30–45°), the elbow flexed (45°), the forearm in a neutral pronation/supination position, and the wrist in ulnar deviation (0–15°) [27]. For the arm assessment, the participant was positioned supine with the head of the bed elevated to 30° to ensure proper upper-limb alignment. The forearms were supinated with palms facing up; pillows were used to stabilise the position as needed [30].

### 2.3. Statistical Analyses

Statistical analyses were performed using *JASP software* (version 0.18.3 for Windows, Amsterdam, the Netherlands). Discrete and continuous data were presented as mean ± standard deviation, *p*-values, mean differences, and confidence intervals. Categorical data were presented as absolute and relative frequencies.

Data normality was assessed using the Shapiro–Wilk test. For non-parametric data, the Wilcoxon signed-rank test was used to compare muscle strength and mean ultrasound measurements between the ipsilateral and contralateral limbs, and the Mann–Whitney U test was used to compare women with lymphoedema and without lymphoedema. For parametric data, paired and independent Student’s *t*-tests were used, respectively. For non-parametric data, the 95% CI was calculated using the Hodges–Lehmann estimator. A *p*-value < 0.05 was considered statistically significant.

Spearman’s correlation test was used to analyse the association between muscle strength variables and the presence and location of lymphoedema. Correlation coefficients (rs) were interpreted according to [31] as weak (rs < 0.3), moderate (0.3 ≤ rs < 0.50), strong (0.5 ≤ rs < 0.70), or very strong (rs ≥ 0.70).

## 3. Results

**Figure 3:**
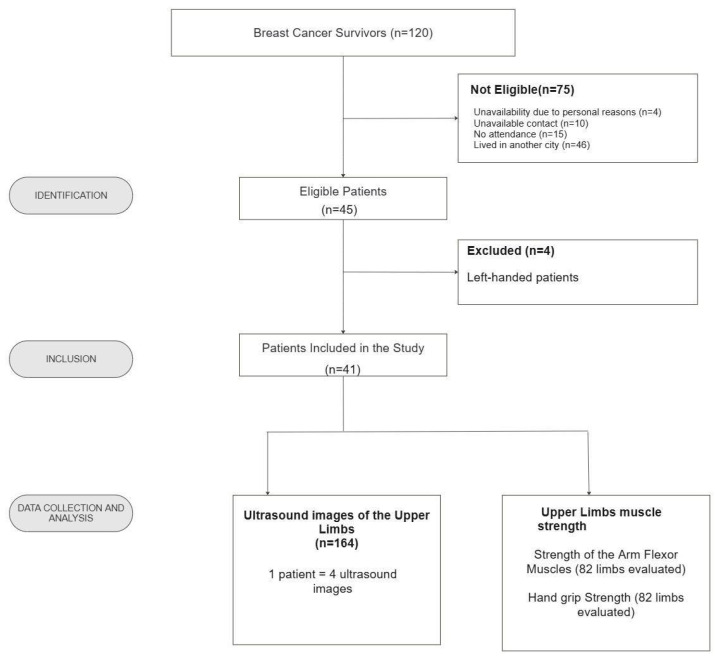
Flowchart of the study screening process, detailing the selection steps for female breast cancer survivors post-mastectomy. Recife/PE, 2022–2023. Source: the authors (2025).

We recruited 120 female breast cancer survivors, of whom 41 were included in this study (Figure 3). In total, we obtained 164 ultrasound images of the upper limbs, representing four per patient (two from the ipsilateral limb and two from the contralateral limb). Muscle strength was assessed using manual and digital dynamometers, resulting in the analysis of 82 upper limbs (ipsilateral and contralateral), with measurements taken separately for each device.

The mean age of the sample was 53.8 ± 7.5 years. The mean time since surgery was 76.2 ± 75.6 months. Regarding the type of surgery, 61.0% underwent a simple mastectomy, 36.5% a radical mastectomy, and 2.5% a modified mastectomy. Regarding lymphoedema, 46.3% of participants had the condition, while 53.7% did not (Table 1).

The mean strength of the arm flexor muscles was significantly lower in the ipsilateral limb (Table 2).

Similarly, peak force was lower in the limb with lymphoedema, with a mean difference of −7.55 (95% CI: −12.25 to −3.18; *p* < 0.001). However, there was no significant difference in time to peak force between the limbs (*p* = 0.936).

Ultrasound analysis revealed a significant increase in the dermal–epidermal complex thickness in the ipsilateral limb, particularly in the forearm (mean difference: 0.20 mm; *p* = 0.022) and arm flexor regions (mean difference: 0.25 mm; *p* < 0.001). Subcutaneous tissue thickness did not differ significantly (*p* = 0.075), whereas muscle thickness was reduced in the forearm (mean difference: −1.80 mm; *p* = 0.002).

As shown in Table 3, the mean strength of the elbow flexor muscles was lower in the lymphoedema group (72.63 ± 24.17) than in the non-lymphoedema group (87.3 ± 51.49). However, the mean difference of 14.72 (95% CI: −11.37 to 40.82) was not statistically significant (*p* = 0.367).

Likewise, absolute handgrip strength did not differ significantly (*p* = 0.628), with a mean of 22.03 ± 5.22 kg in the lymphoedema group and 22.88 ± 5.81 kg in the non-lymphoedema group (mean difference: 0.85 kg; 95% CI: −2.67 to 4.37).

Morphological parameters showed statistically significant differences. The dermal–epidermal complex was thicker in the lymphoedema group in both the forearm (*p* < 0.001) and the arm (*p* < 0.001). Forearm muscle thickness was significantly lower in participants with lymphoedema (9.27 ± 3.22 mm) than in those without it (12.80 ± 4.12 mm; *p* = 0.001).

**Figure 4:**
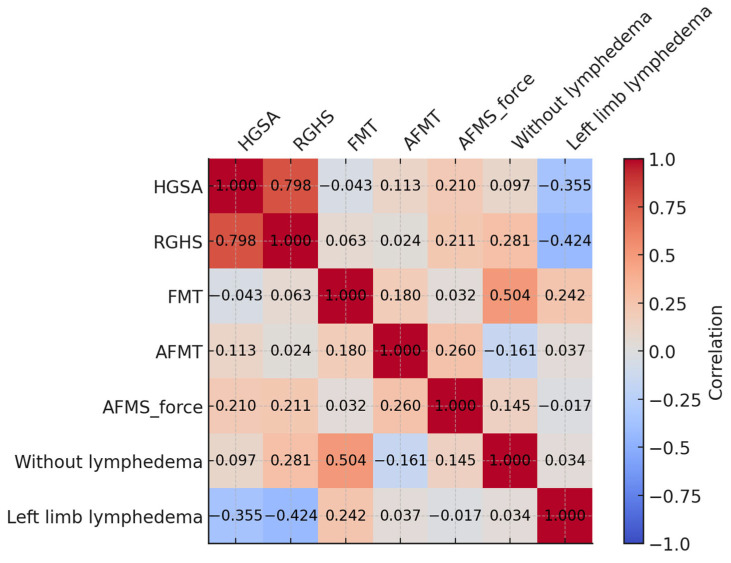
Heatmap of correlation coefficients among the study variables. Caption: AHGS: absolute handgrip strength; RHGS: relative handgrip strength; FMT: forearm muscle thickness; AFMT: arm flexor muscle thickness; AFMS: arm flexor muscle strength. Calculated by Spearman’s correlation test. Source: The authors (2025). Source: the authors (2025).

The heatmap in Figure 4 shows the correlations between the analysed variables. The AHGS and RHGS handgrip strengths were highly correlated (rs = 0.798, *p* < 0.001). The absence of lymphoedema was moderately associated with a higher FMT in the ipsilateral limb (rs = 0.504, *p* < 0.001). Furthermore, a weak negative correlation was found between the presence of lymphoedema in the left (non-dominant) limb and AHGS (rs = −0.335, *p* = 0.023), while a moderate negative correlation was observed with RHGS (rs = −0.424, *p* = 0.006).

## 4. Discussion

This study aimed to analyse the morphofunctional profile of the upper limbs in breast cancer survivors by comparing muscle strength and ultrasound findings between groups with and without lymphoedema and between the ipsilateral and contralateral limbs.

The results demonstrated that the ipsilateral limb had significantly reduced muscle strength, which was associated with the structural changes identified by ultrasound. Although no statistically significant differences in strength were found between the lymphoedema and non-lymphoedema groups, relevant changes in DEC thickness in the arm and forearm, as well as decreased muscle thickness in the forearm, were identified.

The observed decrease in muscle strength in the ipsilateral upper limb indicates functional impairment. This finding aligns with the literature linking the surgical procedure to reduced strength and increased risk of shoulder joint dysfunction, which negatively affect upper limb functionality [32].

Our results showed lower HGS in the ipsilateral limb, corroborating the findings of Campos E Silva et al. [33] and earlier studies reporting reduced strength in breast cancer survivors [34,35]. Higher HGS levels are associated with better functional and psychosocial outcomes, including less disability, pain, and perceived weakness in the affected limb, as well as fewer depressive symptoms. These data reinforce the potential of HGS, both absolute and relative, as a relevant clinical marker for functional and psychosocial monitoring of patients after cancer treatment [21].

Women with breast cancer frequently experience muscle weakness and a decline in physical performance, often associated with increased rest and inactivity, which promotes disuse muscle atrophy and exacerbates functional limitations [36]. HGS assessment is, therefore, a practical and relevant tool for clinical monitoring and functional risk stratification in this patient population.

Although no statistically significant differences in muscle strength were found between the lymphoedema and non-lymphoedema groups, relevant tissue changes were identified. These findings suggest that muscle dysfunction may be a diffuse phenomenon, occurring even in the absence of lymphoedema [37], with evidence of inflammatory processes and systemic structural changes in adjacent tissues [38,39].

The study by [40] supports this view by demonstrating increased collagen deposition and CD4 expression in non-oedematous tissues, which may lead to diffuse impairment of the upper limbs.

Ultrasound enabled a detailed characterisation of structural changes, revealing increased DEC and reduced muscle thickness, primarily in the ipsilateral limb. The lack of consistent differences in subcutaneous tissue thickness may reflect the different stages of lymphoedema. Early stages are characterised by fluid accumulation and increased thickness, while advanced stages can lead to fibrosis and reduced thickness [41]. This coexistence underscores the complexity of diagnosis and clinical monitoring, highlighting the importance of US for precisely assessing the stage and severity of tissue changes.

The lymphoedema group also exhibited increased DEC thickness in the arm and forearm and reduced forearm muscle thickness compared to the non-lymphoedema group. Anatomical and functional factors, such as less efficient lymphatic drainage in the forearm and a predisposition to fluid accumulation in distal areas, contribute to these regional changes. The flatter anatomy of the forearm facilitates ultrasound visualisation of oedema, while the presence of collateral lymphatic pathways in the arm may explain the lower fluid retention in that region [25,42].

Studies suggest that US of the arm flexors, including the biceps brachii, can be a reliable marker of total muscle mass [43], reinforcing its clinical importance. Other studies have also used these muscles and the forearm for similar purposes [44,45], confirming the utility of US in detecting tissue changes associated with BCRL [46,47].

However, despite these advances, a gap remains in the literature regarding studies that integrate assessments of both muscle strength and US-measured tissue thickness in the upper limbs of female breast cancer survivors. In this study, we identified significant correlations between the structural and functional parameters of the upper limbs, including a strong association between absolute and relative handgrip strength and a moderate correlation between the absence of lymphoedema and greater forearm muscle thickness in the ipsilateral limb.

The observed relationship between muscle strength and structure highlights the importance of integrating musculoskeletal ultrasound and dynamometry for functional assessment, enabling a comprehensive and objective analysis of muscle function [48]. The clinical application of US facilitates the early detection of dysfunction, the monitoring of therapeutic responses, and a deeper understanding of the effects of oncology treatments on the underlying tissues [12].

Therefore, our findings support the need for individualised physical therapy strategies that focus on tissue structure, muscle strength, and upper limb functionality, even in the absence of clinically evident lymphoedema. However, this study has limitations, including a lack of control for lymphoedema severity and heterogeneity in post-treatment time, surgery type, and physical activity levels. These variables may influence the functional and structural outcomes, and the lack of statistical control for these factors may have contributed to the observed variations. 

Longitudinal studies are needed to clarify the timeline for functional recovery and the progression patterns of musculoskeletal dysfunction. Moreover, future research should include functional assessments and account for sample heterogeneity in terms of surgery type, treatment duration, age, objective physical activity quantification, and BMI.

## 5. Conclusions

This study revealed significant morphofunctional changes in the upper limbs of breast cancer survivors, particularly between the ipsilateral and contralateral limbs, indicating that oncologic interventions affect both tissue structure and muscle strength. Although no significant differences were found between the lymphoedema and non-lymphoedema groups, the results suggest the need for studies with larger sample sizes and better variable control.

Integrating objective measures of strength and musculoskeletal imaging into physical therapy assessments can improve functional diagnosis, guiding more precise clinical management and personalising rehabilitation strategies. These findings reinforce the importance of continuously monitoring muscle function to prevent complications and promote functional ability and quality of life in this patient population.

## Figures and Tables

**Table 1:** Sociodemographic and clinical characteristics of female breast cancer survivors post-mastectomy (n = 41).

Variable	n (%)	Mean (SD)
Age (years)		53.8 (7.5)
BMI (kg/m^2^)		27.7 (5.0)
Limb with lymphoedema		
Right (dominant)	23 (56.1)	
Left	18 (43.9)	
Time since surgery (months)		76.2 (75.6)
Type of mastectomy		
Simple	25 (61.0)	
Modified	2 (2.5)	
Radical	14 (36.5)	
Therapies received		
Chemotherapy	38 (92.7)	
Radiotherapy	37 (90.2)	
Hormonal	16 (39.0)	
Lymphoedema		
Yes	19 (46.3)	
No	22 (53.7)	

SD: Standard deviation; BMI: body mass index.

**Table 2:** Comparison of muscle strength and ultrasound parameters in the upper limbs of female breast cancer survivors post-mastectomy (n = 41).

Variables	IL Mean (SD)	CL Mean (SD)	*p*-Value	Mean Difference (95% CI)
AFMS				
Mean force (N)	73.23 (25.61)	83.97 (41.85)	<0.001	−5.91 (−9.77 to −2.50)
Peak force (N)	92.84 (34.97)	101.83 (35.21)	<0.001	−7.55 (−12.25 to −3.18)
Time to peak (s)	2.35 (0.53)	2.31 (0.53)	0.936	0.01 (−0.12 to 0.15)
HGS				
AHGS (kg) *	22.48 (5.49)	25.14 (5.83)	<0.001	−2.66 (−4.10 to −1.22)
RHGS (kg/m^2^) *	0.81 (0.24)	0.90 (0.24)	<0.001	−0.09 (−0.15 to −0.04)
US				
FMT (mm)				
DEC	1.40 (1.10)	1.00 (0.20)	0.022	0.20 (<0.01 to 0.55)
SUB	5.25 (2.01)	4.51 (1.87)	0.011	0.65 (0.15 to 1.10)
MT	11.17 (4.04)	12.87(5.13)	0.002	−1.80 (−2.75 to −0.60)
AFMT (mm)				
DEC	1.25 (0.45)	1.02 (0.24)	<0.001	0.25 (0.15 to 0.40)
SUB	6.12 (3.16)	5.79 (2.43)	0.075	0.30 (<−0.01 to 0.60)
MT	15.00 (4.34)	14.18 (5.00)	0.937	2.73 × 10^−5^ (−0.85 to 1.15)

Caption: Data are presented as mean (SD). SD: standard deviation; AFMS: arm flexor muscle strength; HGS: handgrip strength; US: ultrasound; AHGS: absolute handgrip strength; RHGS: relative handgrip strength; FMT: forearm muscle thickness; AFMT: arm flexor muscle thickness; CI: confidence interval; IL: ipsilateral limb; CL: contralateral limb; kg: kilograms; kg/m^2^: kilograms per square meter; MT muscle thickness; DEC dermal–epidermal complex; SUB subcutaneous tissue; mm: millimetres; * calculated using paired Student’s *t*-test.

**Table 3:** Comparison of muscle strength parameters in the ipsilateral limbs between groups with and without lymphoedema (n = 41).

Variables	Lymphoedema (n = 19)	Without Lymphoedema (n = 22)	*p*-Value	Mean Difference (95% CI)
	Mean (SD)	Mean (SD)		
AFMS				
Mean force (N)	72.63 (24.17)	87.36 (51.49)	0.367	14.72 (−11.37 to 40.82)
Peak force (N)	91.98 (33.71)	101.57 (96.03)	0.302	9.59 (−11.25 to 30.44)
Time to peak (s)	2.11 (0.54)	2.43 (0.52)	0.204	0.31 (−0.02 to 0.65)
HGS				
AHGS (kg) *	22.03 (5.22)	22.88 (5.81)	0.628 *	0.85 (−2.67 to 4.37)
RHGS (kg/m^2^) *	0.75 (0.23)	0.87 (0.25)	0.128	0.12 (−0.04 to 0.27)
US				
FMT (mm)				
DEC	1.96 (1.42)	0.94 (0.35)	<0.001	0.70 (0.40 to 1.1)
SUB	5.76 (2.29)	4.77 (1.69)	0.374	0.37 (−0.60 to 2.00)
MT	9.27 (3.22)	12.80 (4.12)	0.001	−3.71 (−5.70 to −0.90)
AFMT (mm)				
DEC *	1.53 (0.46)	1.01 (0.27)	<0.001	0.51 (0.27 to 0.75)
SUB	7.07 (3.94)	5.30 (2.12)	0.084	1.2 (−0.20 to 2.70)
MT *	15.76 (5.39)	14.49 (3.20)	0.357	1.27 (−1.48 to 4.03)

Caption: Data are presented as mean (SD). SD: standard deviation; AFMS: arm flexor muscle strength; HGS: handgrip strength; US: ultrasound; AHGS: absolute handgrip strength; RHGS: relative handgrip strength; FMT: forearm muscle thickness; AFMT: arm flexor muscle thickness; CI: confidence interval; IL: ipsilateral limb; CL: contralateral limb; kg: kilograms; kg/m^2^: kilograms per square meter; mm: millimeters. * calculated using paired Student’s *t*-test.

## Data Availability

The original contributions presented in this study are included in the article. Further inquiries can be directed to the corresponding author(s).

## Abbreviations

The following abbreviations are used in this manuscript:

AHGS	absolute handgrip strength
BCRL	breast cancer-related lymphoedema
BMI	body mass index
DEC	dermal–epidermal complex
RHGS	relative handgrip strength
SCLC	secondary breast cancer lymphoedema
SUB	subcutaneous tissue
FMT	forearm flexors
AFMT	arm flexor muscle thickness
MT	muscle thickness
